# A Novel Tool for the Analysis and Detection of Copy Number Variants Associated with Haemoglobinopathies

**DOI:** 10.3390/ijms232415920

**Published:** 2022-12-14

**Authors:** Anna Minaidou, Stella Tamana, Coralea Stephanou, Maria Xenophontos, Cornelis L. Harteveld, Celeste Bento, Marina Kleanthous, Petros Kountouris

**Affiliations:** 1 Molecular Genetics Thalassaemia Department, The Cyprus Institute of Neurology and Genetics, Nicosia 2371, Cyprus; 2 Human and Clinical Genetics Department, Leiden University Medical Center, P.O. Box 9600, 2333 ZC Leiden, The Netherlands; 3 Department of Haematology, Centro Hospitalar e Universitário de Coimbra, 3000-075 Coimbra, Portugal

**Keywords:** haemoglobinopathies, copy number variants (CNVs), MLPA

## Abstract

Several types of haemoglobinopathies are caused by copy number variants (CNVs). While diagnosis is often based on haematological and biochemical parameters, a definitive diagnosis requires molecular DNA analysis. In some cases, the molecular characterisation of large deletions/duplications is challenging and inconclusive and often requires the use of specific diagnostic procedures, such as multiplex ligation-dependent probe amplification (MLPA). Herein, we collected and comprehensively analysed all known CNVs associated with haemoglobinopathies. The dataset of 291 CNVs was retrieved from the IthaGenes database and was further manually annotated to specify genomic locations, breakpoints and MLPA probes relevant for each CNV. We developed IthaCNVs, a publicly available and easy-to-use online tool that can facilitate the diagnosis of rare and diagnostically challenging haemoglobinopathy cases attributed to CNVs. Importantly, it facilitates the filtering of available entries based on the type of breakpoint information, on specific chromosomal and locus positions, on MLPA probes, and on affected gene(s). IthaCNVs brings together manually curated information about CNV genomic locations, functional effects, and information that can facilitate CNV characterisation through MLPA. It can help laboratory staff and clinicians confirm suspected diagnosis of CNVs based on molecular DNA screening and analysis.

## 1. Introduction

Haemoglobinopathies are the most common monogenic diseases, with millions of carriers and patients worldwide [1]. They are caused by pathogenic variants in the globin gene clusters, which either reduce or abolish the synthesis of globin chains leading to thalassaemia syndromes, or alter the haemoglobin structure leading to structural haemoglobinopathies [2,3]. Over 2300 disease-causing variants have been reported in the IthaGenes database [4]., with their varying severity affecting the clinical manifestations and survival of haemoglobinopathy patients [5].

While most known variants are substitutions and short indels, 291 copy number variants (CNVs) associated with haemoglobinopathies have been reported to date, according to the IthaGenes database. In addition to haemoglobinopathies, the presence of CNVs has been associated with genomic variability and many other genetic disorders. CNVs, mainly large deletions and duplications, within the β-globin gene cluster are relatively rare, causing approximately 10% of all β-thalassaemia cases [6]. CNVs leading to β-haemoglobinopathies involve *HBB* and/or the upstream locus control region βLCR [7]. In contrast to β-haemoglobinopathies, approximately 80–90% of the α-thalassaemia cases are triggered by large genomic deletions/duplications due to meiotic unequal crossovers between misaligned and highly homologous sequence repeats of the α-globin locus [8]. CNVs associated with α-thalassaemia phenotypes affect the α-globin genes and/or the major regulatory element HS-40 of the α-globin locus on chromosome 16 [6,8].

Although asymptomatic carriers and mildly affected patients of the extensive deletional forms of haemoglobinopathies are mainly identified with routine laboratory testing based on haematological and biochemical parameters, their definitive diagnosis requires molecular DNA analysis. Nevertheless, the molecular detection and characterisation of CNVs are challenging and often inconclusive using conventional methods, such as gap-PCR, reverse dot blot (RDB), fluorescent in situ hybridization (FISH) analysis, and Southern blot analysis [6,8]. Alternatively, multiplex ligation-dependent probe amplification (MLPA), in combination with other specific PCR-based methods, has been widely and effectively employed to identify and characterise known, rare, and novel CNVs associated with haemoglobinopathies [6]. The MLPA method can detect large or medium size deletions/duplications, down to a few hundred bases or a single exon. However, data about breakpoints are neither widely available nor easily accessible for all cases and, because of the limitations of the MLPA analysis, the boundaries of several large CNVs are still unclear. The exact genomic breakpoints of a deletion/duplication, following MPLA analysis, require further investigation for complete characterisation, and this is a challenging procedure due to the repetitive regions and highlights of the α- and β-globin loci [9]. However, MLPA is an important tool for grouping chromosomal rearrangements and producing a picture of the known or novel CNVs according to the size and affected genes, including regulatory elements. Accurate identification and structural analysis of CNVs are necessary for the clinical diagnosis of thalassaemia syndromes [10]. Available information on the exact location and effect of previously reported CNVs is often fragmented in the scientific literature or incomplete in different databases, thus further impeding the characterisation and molecular diagnosis of such challenging cases. Consequently, it is often unclear whether several independently reported CNVs are identical or, in fact, novel, while their characterisation through MLPA is challenging due to the lack of knowledge about the exact breakpoints being affected.

Herein, we compile and analyse all the available evidence related to CNVs associated with haemoglobinopathies and we develop an open web tool to facilitate the identification and diagnosis of haemoglobinopathy-specific CNVs. IthaCNVs brings together the available MLPA information and manually curated information available on the ITHANET portal, while providing a user-friendly interface for filtering CNVs based on specific MLPA probes, breakpoints, and affected globin genes and/or regulatory elements.

## 2. Results

**Figure 1:**
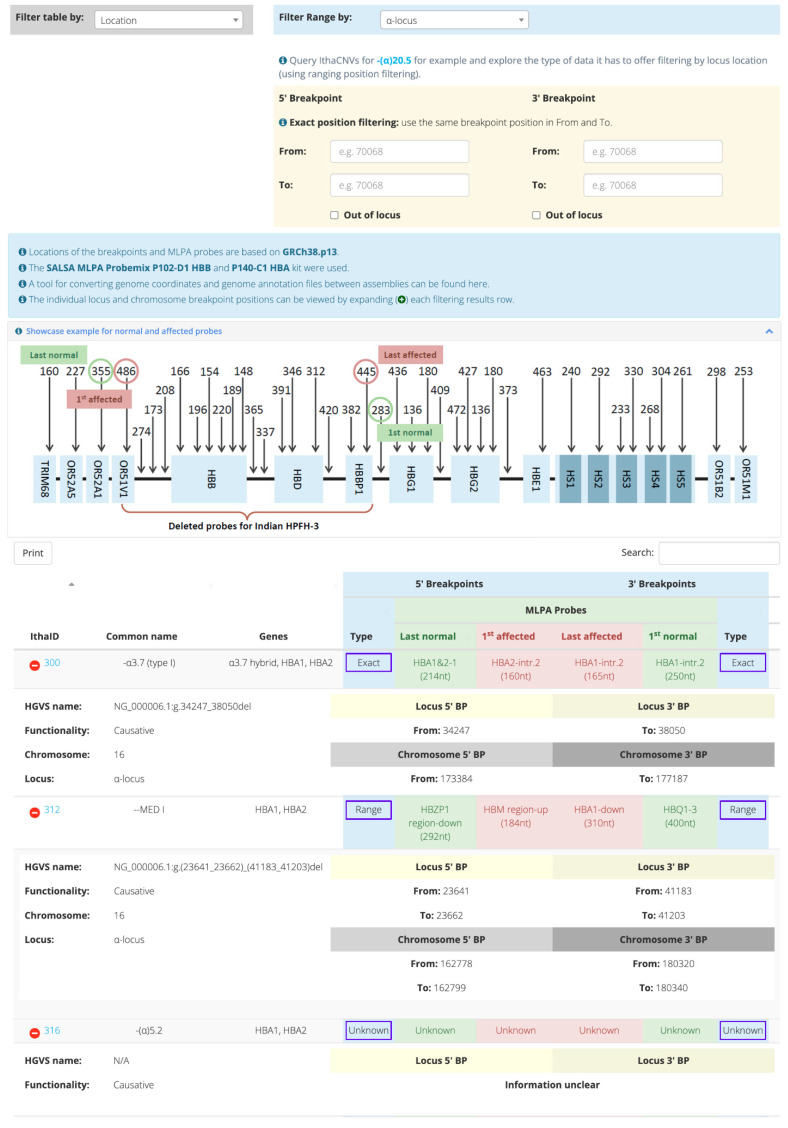
Home page of the IthaCNVs tool. Filtering is based on the CNV location.

Figure 1 illustrates the main functionalities of IthaCNVs, which organises CNV information and facilitates CNV filtering in the following four different ways: (a) according to type of breakpoint information (i.e., the exact location or a range of coordinates harbouring the breakpoint), (b) according to specific locus or chromosomal positions, (c) in relation to MLPA probes, and (d) according to affected gene(s). Initial filtering based on one of four options produces a table with all relevant CNV entries accompanied with further filters acting either on the coordinates or the MLPA probes of the 5′ and 3′ breakpoints, or the affected genes. By expanding the filtering results row, the individual locus and chromosomal breakpoint positions can be displayed. Filtering using the MLPA probes is always based on the chromosomal location from 5′ to 3′ breakpoints, which is the same order that is shown in the ratio chart for the MLPA analysis.

**Figure 2:**
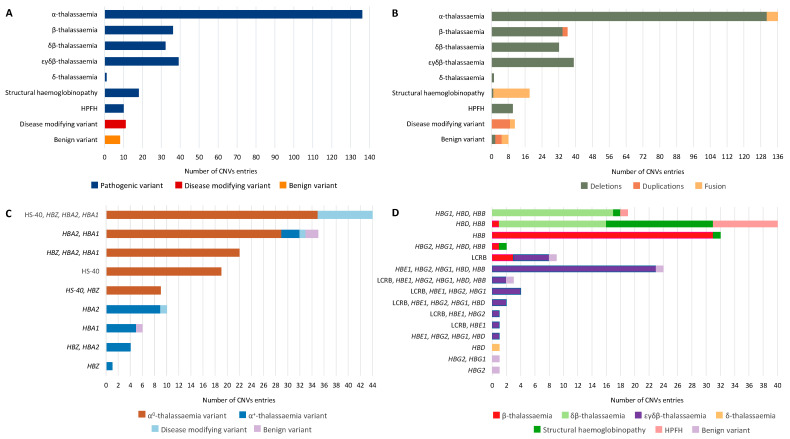
Descriptive analysis of the CNV dataset. (**A**) Haemoglobinopathy groups/phenotypes and variant functionality, (**B**) Haemoglobinopathy groups/phenotypes and variant type, (**C**) Affected genes associated with α-globin locus variants, (**D**) Affected genes associated with β-globin locus variants.

Figure 2A illustrates the distribution of CNVs by according to their associated disease groups/phenotypes, and variant functionality. The dataset comprises 291 CNVs, including 272 pathogenic (93.47%), 11 disease-modifying (3.78%) and 8 benign variants (2.75%). Of the pathogenic CNVs, 136 (50%) are associated with α-thalassaemia, 39 with εγδβ-thalassaemia (14.34%), 36 with β-thalassaemia (13.24%) and 32 with δβ-thalassaemia (11.76%). In rare cases, pathogenic CNVs are associated with a structural haemoglobinopathy (6.62%, 18/272), hereditary persistence of foetal haemoglobin—HPFH (3.68%, 10/272) and δ-thalassaemia (0.37%, 1/272). Notably, one CNV (Hb Jambol; IthaID: 209) can be categorised into two haemoglobinopathy subgroups as the resulting hyperunstable Hb is also characterised by the thalassaemia phenotype [11].

Figure 2B shows the distribution of CNVs based on the variant type and associated disease groups/phenotypes. The CNV dataset contains mainly large deletions (85.91%, 250/291) associated predominantly with thalassaemia phenotypes and HPFH. Of the pathogenic variants, two duplication CNVs are associated with β-thalassaemia (CD 62–83 (+65 bp), IthaID: 2190; >29.5 kb duplication, IthaID: 3869), while five CNVs are associated with α-thalassaemia caused by a fusion of *HBA1*-*HBA2*. Structural haemoglobinopathies are generally associated with the fusion of genes resulting from either a deletion or duplication of a large region within the β-globin locus. Fusion between the different α- or β-globin genes is usually created by an unequal crossing over from the misaligned globin genes, resulting in structurally abnormal haemoglobins [12]. Disease-modifying variants are caused by the duplication and fusion of genes only affecting the α-globin locus (Figure 2C), while benign variants are related with both α- and β-globin loci (Figure 2D).

Importantly, the exact breakpoints on either one or both ends of 26.46% (77/291) of CNVs remain unclear. Because CNVs were either identified by RFLP analysis and/or only recently analysed by MLPA, the precise breakpoints were not captured. Therefore, the 3′ and 5′ breakpoints of these CNVs are characterised as a range, since they have not been sequenced. For these CNVs, GAP-PCR and Sanger sequencing can be performed for the exact specification of the breakpoints. In some cases, the 3′ or the 5′ end of the CNVs is not defined because of the limitation of the technique used. A common problem in characterising CNVs affecting the globin gene loci is that one or both breakpoints fall out of the range of MLPA probes. In cases where no additional techniques were used, the exact breakpoints beyond the probe locations remain unclear. When breakpoints extend beyond the MLPA probes, the size and approximate breakpoint localisation can be determined by performing an array CGH analysis [13].

Furthermore, and despite rigorous research, 9.27% (27/291) of all the CNVs presented in this study have unknown breakpoints due to limited information. Since some of the CNVs have only been reported in a single case study or have been identified in the context of other diseases, there is limited information available to clarify their breakpoints. The information provided reflects only the name of the deleted genes or the approximated size of the CNV. In addition, information about CNVs reported only once and around 30–40 years ago is often limited and inconclusive regarding the exact location.

Figure 2C and Supplementary Figure S1 focus on CNVs affecting the α-globin locus. A schematic representation of all CNVs along with the corresponding locations of α-globin cluster on chromosome 16, is presented in Supplementary Figure S1. Supplementary Figure S1A presents the CNVs restricted within the α-globin locus (31.91%) and the specifically affected genes. As illustrated in Supplementary Figure S1, the majority of CNVs associated with α-globin locus variants (68.09%) are very large CNVs extending beyond the α-globin locus leading to α^0^-thalassaemia. Among the pathogenic α-globin locus variants, 83.82% (114/136) are related to large deletions affecting both *HΒA1*, *HΒA2* and/or HS-40 leading to α^0^-thalassaemia, with the remaining 16.18% (22/136) causing defects in either *HΒA1* or *HΒA2* and resulting in α^+^-thalassaemia (Figure 2C)—except for four deletions (-α3.7 (type I), IthaID: 300; -α3.7 (type II), IthaID: 2230; -α3.7 (type III), IthaID: 2231; and α12, IthaID: 2555) which, despite extending within *HΒA1* and *HΒA2* (Supplementary Figure S1A)*,* create a single functional fusion-gene leading to α^+^-thalassaemia. Despite affecting *HΒA2*, *HΒA1* and/or HS-40, and part of the telomeric region of chromosome 16, as illustrated in Supplementary Figure S1B, the disease-modifying variants (α-globin locus duplication variants) are caused by large duplications or triplications of α-globin genes and do not usually lead to pathogenesis, unless they are combined with β-thalassaemia alleles. Benign variants are created by deletions or fusions that do not alter the functionality of *HBA1* and *HBA2.*

Figure 2D and Supplementary Figure S2 focus on CNVs affecting the β-globin locus. A related schematic representation of all CNVs, along with the corresponding locations of β-globin cluster on chromosome 11, is presented in Supplementary Figure S2. There is no significant difference in the number of CNVs restricted within the β-globin locus (56.20%, Supplementary Figure S2A) and larger CNVs extending beyond the β-globin locus (43.80%, Supplementary Figure S2B). According to Figure 2D, pathogenic β-globin locus CNVs are divided into the following six groups: δβ-thalassaemia (23.53%, 32/136), β-thalassaemia (26.47%, 36/136), εγδβ-thalassaemia (28.68%, 39/136), δ-thalassaemia (0.74%, 1/136), structural haemoglobinopathies (13.24%, 18/136) and HPFH (7.35%, 10/136). CNVs associated with εγδβ-thalassaemia represent the largest deletions that disturb the entire β-globin locus (59%, 23/39) including *HBB*, *HBD*, *HBG1*, *HBG2*, *HBE1* and βLCR. Notably, the locus control region βLCR is affected in almost all CNVs (92.31%, 36/39) associated with εγδβ-thalassaemia, demonstrating the important role of βLCR in the gene expression of the β-globin locus. CNVs associated with the δβ-, β-, and δ-thalassaemia are smaller deletions that affect both *HBB* and *HBD*, or only *HBB* or *HBD*, respectively. Exceptions include five CNVs that cause β-thalassaemia. In the first three cases (1992 bp deletion, IthaID: 2479; Caribbean, IthaID: 2566; 12.4 kb Mediterranean deletion, IthaID: 3935), the deletions affect a limited region of βLCR, as illustrated in Supplementary Figure S2A. The deletion involves only one or two (HS3 or/and HS4) of the five DNase I hypersensitivity (HS) regions of βLCR, which only impairs the expression of *HBB,* leaving the expression of the remaining genes of the β-globin locus intact [14]. Moreover, another CNV (223 kb deletion; IthaID: 3394) does not cause δβ-thalassaemia, despite affecting both *HBB* and *HBD*, because it extends from the 3′UTR of the *HBD* without affecting its expression [15]. The last CNV (>29.5 kb duplication; IthaID: 3869) is a duplication including *HBG2*, *HBG1*, *HBD,* and part of *HBB*. Therefore, only *HBB* is disturbed, resulting in β-thalassaemia. Structural haemoglobinopathies are caused by CNVs affecting *HBD* and *HBB*, leading to a fusion of the genes, which gives rise to abnormal hybrid haemoglobin variants. Three of the five benign variants are duplications of the entire β-globin locus (African I duplication, IthaID: 2550; African II duplication, IthaID: 3112) or the *HBG2* (Gγ duplication, IthaID: 3596), while the fourth is a deletion (Italian HS1, IthaID: 3936) involving only the HS1 region of the βLCR (Supplementary Figure S2A) leaving the functionality of βLCR and β-globin genes intact [16]. The last benign variant is a deletion involving part of *HBG1* and *HBG2*, leading to a single functional fusion-gene.

## 3. Discussion

The present study is the most comprehensive analysis of haemoglobinopathy-associated CNVs to date. All available CNV information related to haemoglobinopathies was investigated and analysed, and an open tool that facilitates the identification and diagnosis of these CNVs was developed.

The importance of fully characterising CNVs associated with haemoglobinopathies is still as relevant today as it was decades ago when identification and characterisation of large deletion/duplications first began. The combination of a rare CNV removing both *HBA1* and *HBA2* with more prevalent α-globin gene pathogenic variants can result in clinically significant conditions, such as Hb H disease and the usually lethal Hb Bart’s hydrops fetalis syndrome.

Furthermore, HPFH and δβ-thalassaemia are molecularly similar conditions located at the end of the β-globin locus involving the *HBB* and *HBD*. As both conditions are characterised by elevated foetal haemoglobin (HbF) levels, the distinction between them is based on phenotypic differences and haematological indices. Heterozygotes of HPFH are characterised by normal red cell indices, normal HbA2 level and increased levels of HbF (15–30%) with homogeneous pancellular distribution, while δβ-thalassaemia heterozygotes are characterised by hypochromic and microcytic red cells, a normal level of HbA2 and a less severe increase in HbF levels (5–15%) with heterogeneous intercellular distribution [17,18]. In addition, the compound heterozygosity of an HPFH deletion with a β^0^-thalassaemia mutation can lead to an asymptomatic condition involving mild anaemia [19]; this is in contrast to the effect of compound heterozygosity of δβ-thalassaemia along with another form of β-thalassaemia that leads to a severe clinical picture and a transfusion-dependent condition [18,20]. Most cases of HPFH and δβ-thalassaemia are sporadic and rare; therefore, further evaluation and additional data are required for adequate categorisation. A complex network of factors and regulatory regions is known to affect the HbF levels which, along with the genetic characteristics that patients carry, favour a higher level of HbF expression [21]. Clarifying the breakpoints of deletions leading to either δβ-thalassaemia or HPFH will shed light on the molecular mechanisms that enhance γ-globin production and the elevation of HbF levels [18]. For example, two CNVs that delete the entire *HBB* (South East Asian (SEA) deletion; IthaID: 1505 and Caucasian HPFH. IthaID: 2124) present a typical β-thalassaemia trait phenotype in the heterozygous state with microcytic, hypochromic erythrocytes and increased HbA2 level, but also with an elevated level of HbF. These two large deletions (>27 kb) were first characterised as HPFH because of significantly raised HbF levels of over 20%. Identification of the exact breakpoints in recent studies indicated that the deletions are more akin to β-thalassaemia syndrome with elevated HbF, as the 3′ breakpoint downstream of the β-globin gene was found to contain the HS-1 γ-silencing element, while *HBD* and erythroid-specific elements at the 5′ breakpoint remained intact [22,23,24]. Considering the above deletions of HPFH, misleading genetic counselling for a clinically mild condition could occur in cases of compound heterozygosity with β^0^-thalassaemia mutation. Therefore, complete characterisation and the easier detection and identification of CNVs is important not only for the correct diagnosis and treatment of the patients, but also for genotype–phenotype correlation and for comprehensive prenatal genetic counselling of the carriers.

Another characteristic example is εγδβ-thalassaemia, a rare thalassaemia syndrome caused by large deletions affecting the β-globin gene cluster. To date, over 30 such deletions have been described in heterozygous form only [25]. In general, homozygous deletions affecting the entire β-globin gene cluster are very rare presumably because they are not compatible with foetal survival [7]. Severe neonatal haemolytic anaemia is a characteristic clinical manifestation of εγδβ-thalassaemia, with affected neonates distinguished from β-thalassaemia patients due to the requirement of intrauterine and often perinatal red blood cell transfusions until the age of six months old. Beyond the first few months of life, anaemia is spontaneously normalised due to the elevated expression and production of β-globin in the first year of life [25,26]. Adult heterozygote haematological parameters resemble the β-thalassaemia trait phenotype, but due to normal HbA2 levels, εγδβ-thalassaemia is commonly undiagnosed or misdiagnosed [27]. Furthermore, hypochromic and microcytic indices with normal HbA2 levels could be due to compound heterozygous variants in *HBD* and *HBB*, as well as in α^0^-thalassaemia carriers, leading to a differential diagnosis of εγδβ-thalassaemia [25]. Without proper testing, the diagnosis may be overlooked, and the patient may be improperly treated.

Furthermore, determining the exact breakpoints of CNVs can lead to delineation of novel regulatory regions and other genes related with the expression of the α and β-globin clusters. Intergenic enhancer and repressor, miRNAs, non-coding RNAs, transcriptional and epigenetic regulators, and epigenetic factors, may regulate the suppression and activation of globin genes as found in other genes in human cells [18,19]. Moreover, many CNVs trigger human genetic diseases associated with developmental disabilities [28]. In thalassaemia syndromes, CNVs are associated with the occurrence and severity of the disease [13]. Additionally, a comparison of different CNV breakpoints can provide insights into the biological processes that create them.

The MLPA technique has been proven to identify the structure and estimate the size of the arrangement region of known and unknown CNVs in unsolved cases after performing conventional techniques. MLPA is routinely used as a diagnostic screening tool in many laboratories, for many hereditary and acquired disorders triggered by gene dosage changes, and requires only a thermocycler and capillary electrophoresis equipment [22,29]. Nevertheless, the MLPA method can omit small deletions as the probes are not continuous or overlapping. In addition, the distance between many MLPA probes ranges between 3 and 10 kb; hence, if the CNV breakpoints extend beyond the range of MLPA probes, the use of array CGH analysis is required for size and localisation determination and also the use of GAP-PCR/CBAS and DNA sequencing for complete identification of the breakpoints [13,30]. The whole procedure is time-consuming and labour-intensive, making the characterisation of unknown CNVs highly complicated. Moreover, the presence of large repetitive sequences, which characterise both α- and β-globin loci, makes the identification of CNV breakpoints even more difficult as the repetitive sequences restrict the efficiency of the methods [31].

The majority of the CNVs are extremely rare and unique to the families in which they have been described. As more of such CNVs are identified with universal screening, there is a need for a comprehensive single methodology akin to next-generation sequencing that can fully characterise breakpoints and genomic recombination events in a single analytical process [32]. Many studies suggest that WGS can be used to accurately locate the breakpoints of large segment deletions/duplications [33]. Furthermore, a recent study presented the development of a CNV application based on quantitative real-time PCR (qPCR) for the suspected cases of α-thalassaemia following normal gap-PCR results [34].

Due to the high percentage of genomic deletions involving the α-globin gene cluster and the high heterogeneity of the deletions within the β-globin cluster, the IthaCNVs tool can facilitate laboratory staff and clinicians to confirm the suspected diagnosis of CNVs based on molecular DNA screening and analysis. Although in some cases the pathogenicity of a CNV can be inferred from the molecular analysis (e.g., CNVs removing both *HBA1* and *HBA2* causing α-thalassaemia), the interpretation of CNVs should combine evidence about the disease mechanism, phenotype, and mode of inheritance, as suggested by the ACMG/AMP guidelines. Fully integrated with other ITHANET databases, IthaCNVs provides a manually curated list of all known CNVs associated with haemoglobinopathies and, through its diverse filtering options, it can facilitate the diagnosis of rare and diagnostically challenging cases and can potentially lead to standardised classification of such cases [35,36].

## 4. Materials and Methods

The list of all CNVs (Supplementary File S1) associated with haemoglobinopathies was retrieved from the IthaGenes database (Access Date: 7 November 2022). Specifically, the dataset of this study includes 291 CNVs (>50 bp), including pathogenic, disease-modifying, and benign variants. CNVs with known breakpoints were reviewed to either confirm or correct the exact location of the upstream (5′) and downstream (3′) breakpoints using available evidence and the latest literature, while a rigorous literature review was performed for CNVs with missing breakpoint information. In many cases, MLPA was the only method that was used for CNV clarification, resulting in breakpoint locations between 5′ and 3 instead of the exact genomic locations. This range is the gap between the two probes in which the breakpoint occurred. At the 5′ breakpoint, the first genomic location of the range is not affected and represents the last normal amplified probe, while the second location is affected and represents the first affected probe. At the 3′ breakpoint, the first genomic location of the range is affected and represents the last affected probe, while the second location is not affected and represents the first normal amplified probe (Figure 1). In addition, the breakpoints of some CNVs reported in the past were not available because they were either characterised with restriction fragment length polymorphism (RFLP) analysis or identified during screening for other disorders and only the relevant region or deleted genes were reported. The updated CNV dataset was subsequently independently reviewed by three diagnostic laboratories from Cyprus, the Netherlands, and Portugal, with members co-authoring this study, to help identify discrepancies and provide the missing information. Subsequently, the available MLPA probes involved in each CNV were identified using information about their positions and names from the MRC-Holland commercial kits (https://www.mrcholland.com/) for both globin loci, accessed on 7 November 2022. The SALSA MLPA Probemix P140-C1 HBA and P102-D1 HBB kit were used for the α- and β-globin locus, respectively.

We developed IthaCNVs (https://ithanet.eu/db/ithacnv) using the existing infrastructure of the ITHANET portal based on PHP/MySQL, as described by Kountouris et al., 2014 [4]. All CNVs are linked to their corresponding variant page on the IthaGenes database, where additional details about associated phenotypes, context sequence, external links and relevant publications are provided.

## Data Availability

IthaCNVs and the associated data are freely available on the ITHANET portal at https://ithanet.eu/db/ithacnv.

## Supplementary Materials

The following supporting information can be downloaded at: https://www.mdpi.com/article/10.3390/ijms232415920/s1.
