# Supplementary material for: A Novel Tool for the Analysis and Detection of Copy Number Variants Associated with Haemoglobinopathies
Source: Int J Mol Sci. 2022 Dec 14;23(24):15920. doi: 10.3390/ijms232415920 (PMC9782104; doi:10.3390/ijms232415920)
Supplement: Supplementary file 1 [file ijms-23-15920-s001.zip › Supplementary Figure S1.pdf]

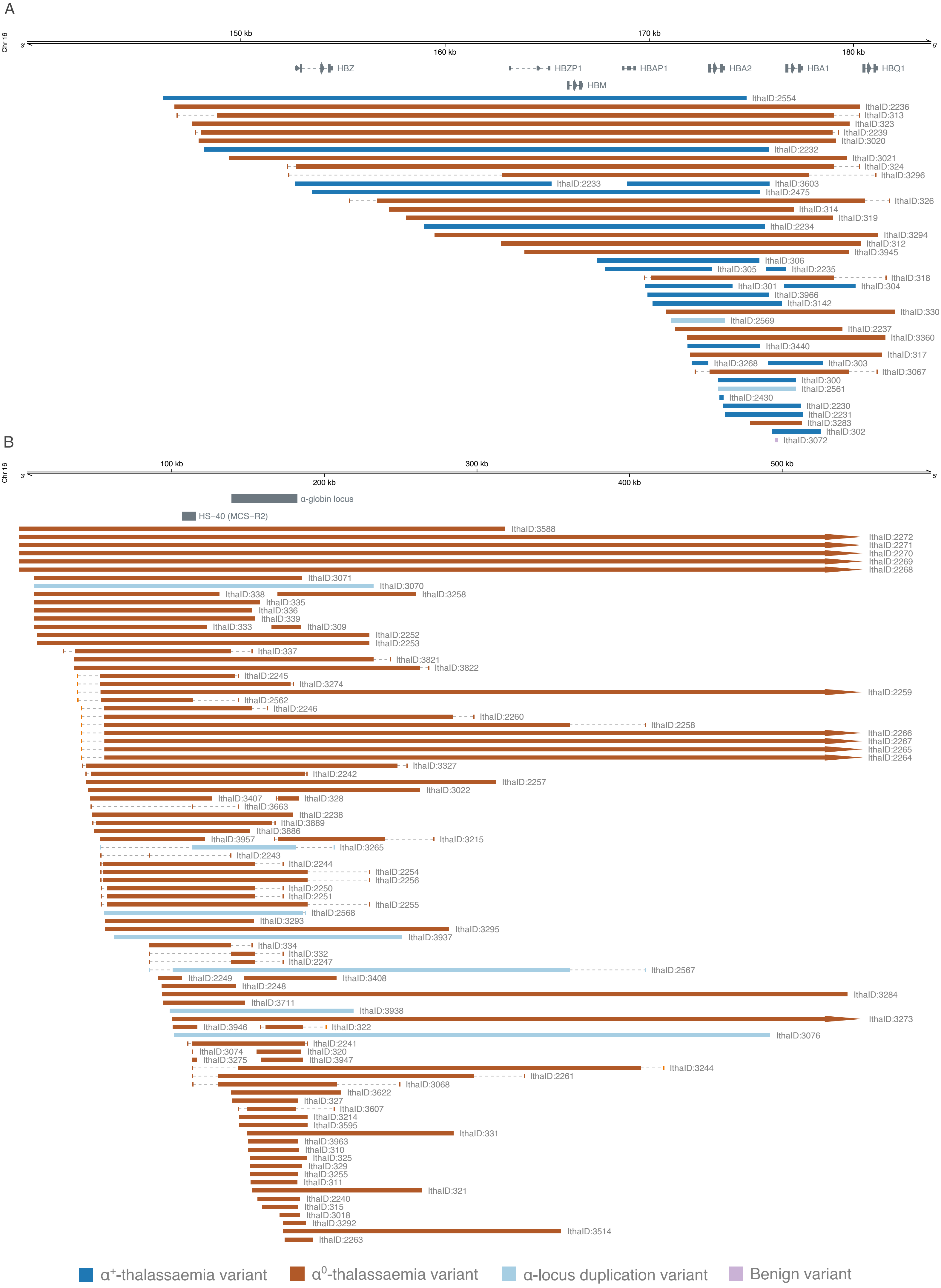
A
Chr 16
3'
5'
150 kb
160 kb
170 kb
180 kb
HBZ
HBZP1
HBAP1
HBA2
HBA1
HBQ1
HBM
IthaID:2554
IthaID:2236
IthaID:313
IthaID:323
IthaID:2239
IthaID:3020
IthaID:2232
IthaID:3021
IthaID:324
IthaID:3296
IthaID:2233
IthaID:3603
IthaID:2475
IthaID:326
IthaID:314
IthaID:319
IthaID:2234
IthaID:3294
IthaID:312
IthaID:3945
IthaID:306
IthaID:305
IthaID:2235
IthaID:318
IthaID:301
IthaID:304
IthaID:3966
IthaID:3142
IthaID:330
IthaID:2569
IthaID:2237
IthaID:3360
IthaID:3440
IthaID:317
IthaID:3268
IthaID:303
IthaID:3067
IthaID:300
IthaID:2561
IthaID:2430
IthaID:2230
IthaID:2231
IthaID:3283
IthaID:302
IthaID:3072
B
Chr 16
3'
5'
100 kb
200 kb
300 kb
400 kb
500 kb
α-globin locus
HS-40 (MCS-R2)
IthaID:3588
IthaID:2272
IthaID:2271
IthaID:2270
IthaID:2269
IthaID:2268
IthaID:3071
IthaID:3070
IthaID:338
IthaID:3258
IthaID:335
IthaID:336
IthaID:339
IthaID:333
IthaID:309
IthaID:2252
IthaID:2253
IthaID:337
IthaID:3821
IthaID:3822
IthaID:2245
IthaID:3274
IthaID:2259
IthaID:2562
IthaID:2246
IthaID:2260
IthaID:2258
IthaID:2266
IthaID:2267
IthaID:2265
IthaID:2264
IthaID:3327
IthaID:2242
IthaID:2257
IthaID:3022
IthaID:3407
IthaID:328
IthaID:3663
IthaID:2238
IthaID:3889
IthaID:3886
IthaID:3957
IthaID:3215
IthaID:3265
IthaID:2243
IthaID:2244
IthaID:2254
IthaID:2256
IthaID:2250
IthaID:2251
IthaID:2255
IthaID:2568
IthaID:3293
IthaID:3295
IthaID:3937
IthaID:334
IthaID:332
IthaID:2247
IthaID:2567
IthaID:2249
IthaID:3408
IthaID:2248
IthaID:3284
IthaID:3711
IthaID:3938
IthaID:3273
IthaID:3946
IthaID:322
IthaID:3076
IthaID:2241
IthaID:3074
IthaID:320
IthaID:3275
IthaID:3947
IthaID:3244
IthaID:2261
IthaID:3068
IthaID:3622
IthaID:327
IthaID:3607
IthaID:3214
IthaID:3595
IthaID:331
IthaID:3963
IthaID:310
IthaID:325
IthaID:329
IthaID:3255
IthaID:311
IthaID:321
IthaID:2240
IthaID:315
IthaID:3018
IthaID:3292
IthaID:3514
IthaID:2263
α+-thalassaemia variant
α0-thalassaemia variant
α-locus duplication variant
Benign variant
