# Supplementary figures and images for: A Novel Tool for the Analysis and Detection of Copy Number Variants Associated with Haemoglobinopathies
Source: Int J Mol Sci. 2022 Dec 14;23(24):15920. doi: 10.3390/ijms232415920 (PMC9782104; doi:10.3390/ijms232415920)

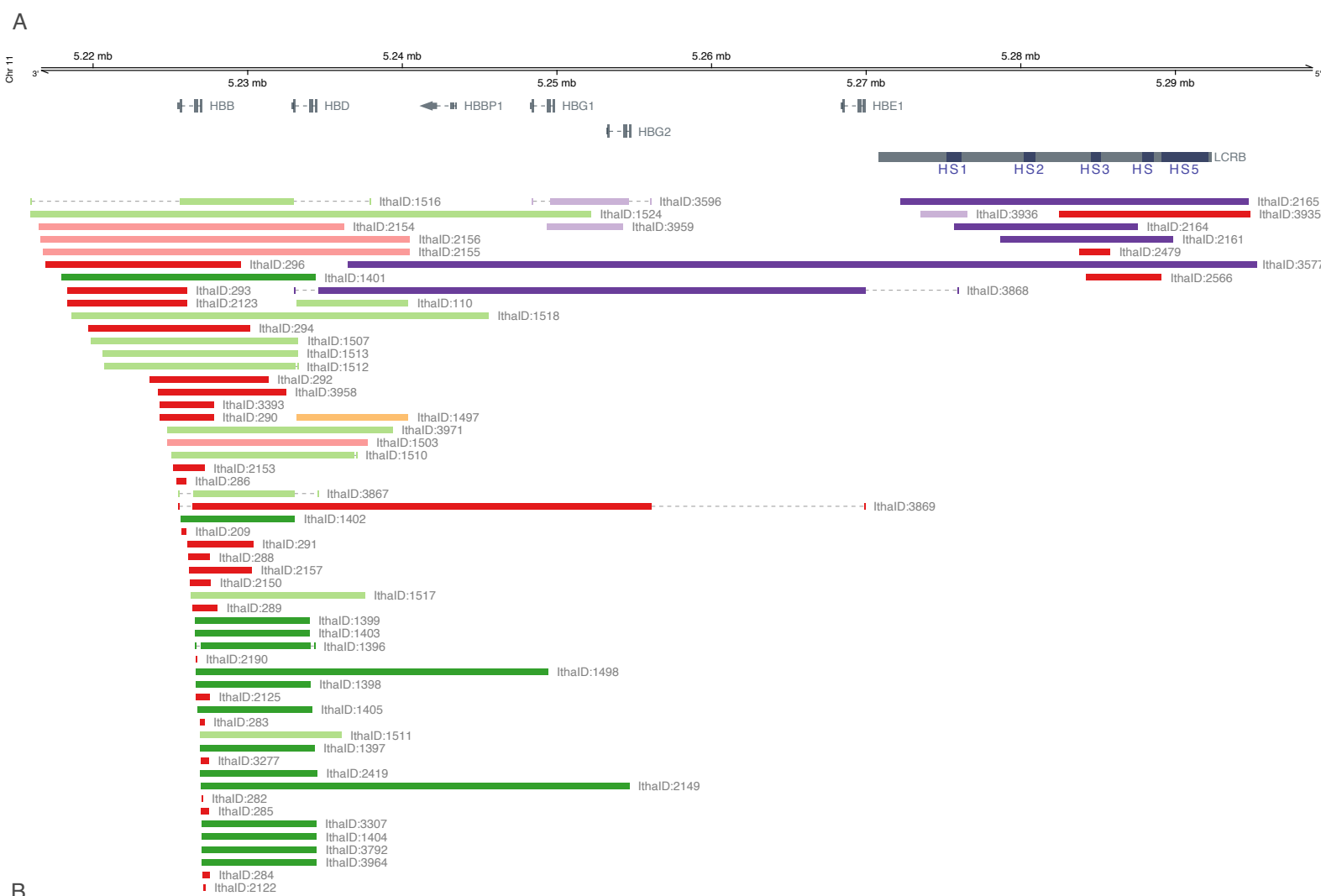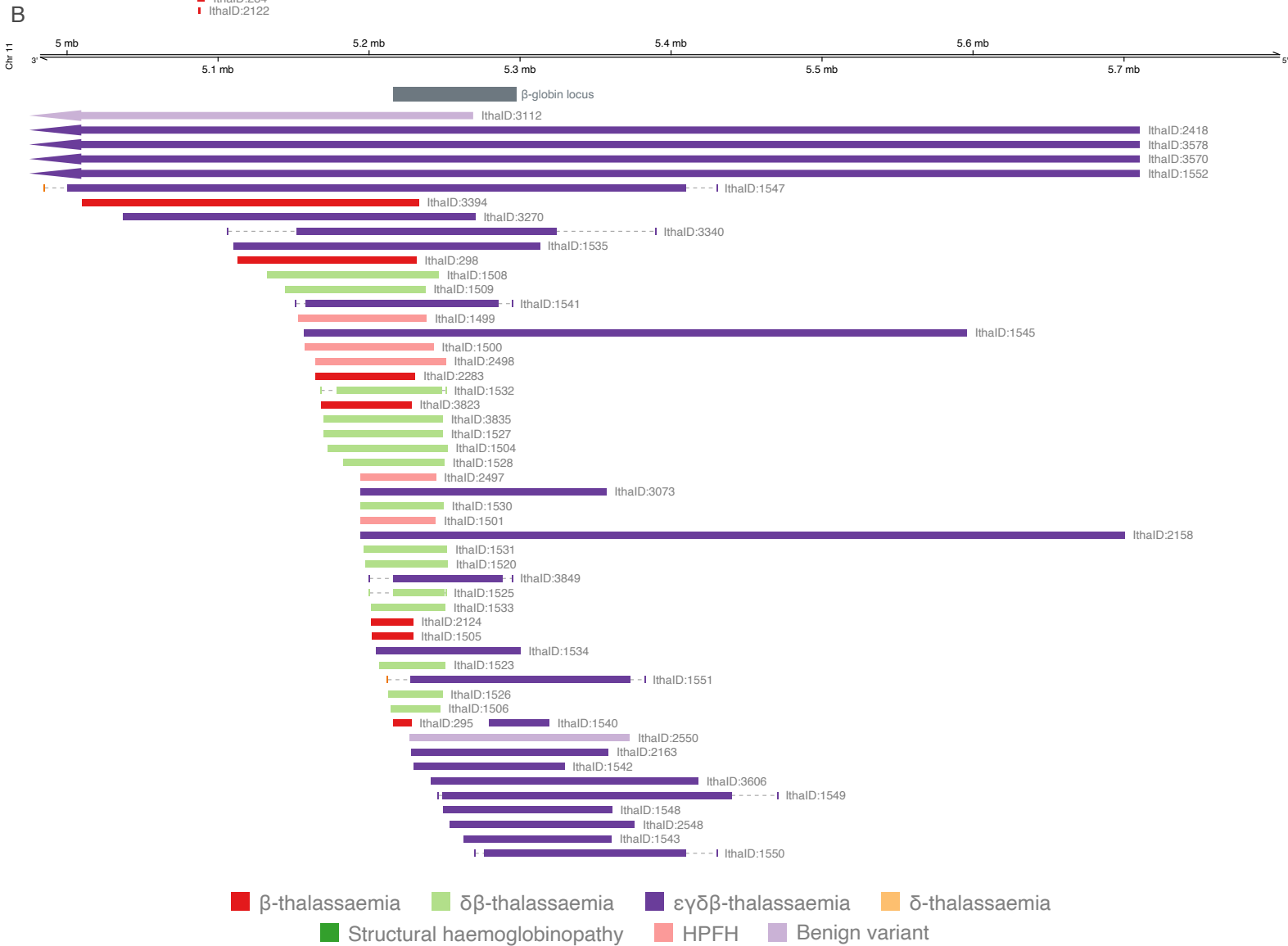

Supplement: Supplementary file 1 [file ijms-23-15920-s001.zip › Supplementary Figure S2.pdf]
